# Irradiation-Induced Changes in the Immunogenicity of Lung Cancer Cell Lines: Based on Comparison of X-rays and Carbon Ions

**DOI:** 10.3389/fpubh.2021.666282

**Published:** 2021-04-22

**Authors:** Juntao Ran, Jiangtao Wang, Ziying Dai, Yandong Miao, Jian Gan, Chengpeng Zhao, Quanlin Guan

**Affiliations:** ^1^Department of Radiation Oncology, The First Hospital of Lanzhou University, Lanzhou, China; ^2^The First Clinical Medical College of Lanzhou University, Lanzhou, China; ^3^Department of Oncology, The First Hospital of Lanzhou University, Lanzhou, China; ^4^Department of Oncology Surgery, The First Hospital of Lanzhou University, Lanzhou, China

**Keywords:** irradiation, immunogenicity, lung cancer, X-rays, carbon ions

## Abstract

Increasing the immunogenicity of tumors is considered to be an effective means to improve the synergistic immune effect of radiotherapy. Carbon ions have become ideal radiation for combined immunotherapy due to their particular radiobiological advantages. However, the difference in time and dose of immunogenic changes induced by Carbon ions and X-rays has not yet been fully clarified. To further explore the immunogenicity differences between carbon ions and X-rays induced by radiation in different “time windows” and “dose windows.” In this study, we used principal component analysis (PCA) to screen out the marker genes from the single-cell RNA-sequencing (scRNA-seq) of CD8^+^ T cells and constructed a protein-protein interaction (PPI) network. Also, ELISA was used to test the exposure levels of HMGB1, IL-10, and TGF-β under different “time windows” and “dose windows” of irradiation with X-rays and carbon ions for A549, H520, and Lewis Lung Carcinoma (LLC) cell lines. The results demonstrated that different marker genes were involved in different processes of immune effect. HMGB1 was significantly enriched in the activated state, while the immunosuppressive factors TGF-β and IL-10 were mainly enriched in the non-functional state. Both X-rays and Carbon ions promoted the exposure of HMGB1, IL-10, and TGF-β in a time-dependent manner. X-rays but not Carbon ions increased the HMGB1 exposure level in a dose-dependent manner. Besides, compared with X-rays, carbon ions increased the exposure of HMGB1 while relatively reduced the exposure levels of immunosuppressive factors IL-10 and TGF-β. Therefore, we speculate that Carbon ions may be more advantageous than conventional X-rays in inducing immune effects.

## Introduction

Radiotherapy (RT) is the primary treatment for lung cancer, which first-line treatment accounts for ~30% of all newly diagnosed patients (1). Nevertheless, lung cancer treatment is still tricky, and a new treatment method is urgently needed (2). Relevant studies have shown that immunotherapy has a positive impact on the treatment endpoint of lung cancer and has changed lung cancer treatment. Immunotherapy has become the most promising and effective treatment for lung cancer (3). The effectiveness of RT is explained as reasonable local tumor control and practical immune activation effect (4, 5). However, in addition to activating immunity, radiation also has an immunosuppressive effect (6), including the recruitment or polarization of immunosuppressive cytokines, immune checkpoint molecules, and suppressive immune cell subtypes (7). Therefore, it is necessary to understand the immunomodulatory properties of radiation to enhance the immune synergy of radiotherapy. Carbon ions have significant radiobiological advantages over conventional X-rays (8, 9), and the direct killing effect on radiation-resistant tumor cells is stronger than conventional X-rays 2-3 times (5, 10). Therefore, it is essential to analyze the immunogenic changes induced by two kinds of radiation in tumor cells to improve radioimmunity.

Immunogenic cell death (ICD) is a form of cell death that can be recognized by the immune system and induce a specific anti-tumor immune response (11). ICD relies on the specific stimuli while provoking the temporal and spatial coordinated immunogenic signals (12, 13), including tumor-associated antigens (TAAs) and damage-associated molecular patterns (DAMPs) related to the activation of dangerous signals pathways (14, 15). Various stimuli, including radiotherapy, chemotherapy, and oncolytic viruses (OVs), can induce ICD (16, 17). In theory, the advantage of enhancing ICD is that it can stimulate the immune system.

CD8^+^T cells are the primary effector cells involved in the anti-tumor immune response, especially during the immune response process caused by radiation (18). The T cell receptor (TCR) on the surface of T cells binds to the antigen-MHC complex and then establishes the immune response by clonal expansion (19). Due to the heterogeneity and difference between cells, the expression of differential genes in CD8^+^ T cells may play different roles in the process of participating in immune effects in the activated or resting state (20). RT combined with immunotherapy has become an effective treatment for NSCLC (21, 22). The immune regulation mechanism induced by radiation has also become an essential aspect of forming the abscopal effect and improving the prognosis. However, the effect of different radiation on the immune response is poorly understood. The change of tumor immunogenicity induced by radiation is an essential mechanism for improving tumor microenvironment (TME) and immune synergism (23). Among them, HMGB1, TGB-β, and IL-10 are important cytokines in radiation-induced ICD and then participate in the process of immune regulation (24–26). As one of the criticalcrucial DAMPs, HMGB1 plays an essential role in the immune effect stage (27). As classic immunosuppressive factors, the increase of TGB-β and IL-10 is often accompanied by immune effector cell function inhibition and the increase of tumor-associated macrophage (TAMs) infiltration (28, 29).

In this study, based on the scRNA-seq results of CD8^+^T cell clusters in lung cancer tissues in the GEO database, we performed PCA and Cluster analysis on the distribution of the differential genes, which were screened out by bioinformatics methods. What is essential, we compared the effects of X-rays and carbon-ions radiation on the change trends of HMGB1, IL-10, and TGF-β under different doses and times by ELISA and further explored the role in the immune process of CD8^+^ T cells.

## Materials and Methods

### Data Retrieval and Processing

In this research, scRNA-seq of CD8^+^ T Cell clusters isolated from human lung and lung tumor samples with flow cytometry was downloaded from Gene Expression Omnibus (GEO, https://www.ncbi.nlm.nih.gov/geo/) datasets. GSE111894, with 1,084 human lung samples and GPL16791 platform, was selected (30).

### Principal Component Analysis (PCA), TSNE Cluster Analysis, and Marker Gene Annotation

Use R language to perform PCA dimensionality reduction processing on the downloaded scRNA-seq data of lung cancer CD8^+^T cell clusters and screen out the relevant genes of each principal component. On this basis, perform TSNE cluster analysis and visualization to find differential expression genes (DEGs) and draw the scatter diagram and violin diagram of the marker genes in each cluster.

### Construction of Protein-Protein Interaction Network and Protein Co-Expression Analysis

The marker genes of the different principal components were submitted to the STRING database (http://www.string-db.org/) to clarify the information of protein-protein interaction (PPI) (31). The protein co-expression network was constructed and visualized by Cytoscape 3.7.1 software. The number of nodes adjacent to each protein was calculated and sorted by the Molecular Complex Detection (MCODE) plug-in with an MCODE score of more than two (32). Besides, the selected high-risk proteins were analyzed for protein co-expression and visualization (33). *P* < 0.05 was considered to have statistical significance.

### Cell Lines

Human LUAD cell lines A549 and LUSC cell lines NCI-H520 were purchased from the Cell Bank, Type Culture Collection, Chinese Academy of Sciences (CBTCCCAS). Mouse Lewis lung cancer cells (LLC) was purchased from Cellcook Co, Ltd, Guangzhou, China. Cells were cultured in Dulbecco's Modified Eagle Medium (GIBCO, US) containing 10% fetal bovine serum (BIOWEST, France) and 1% penicillin/streptomycin (HyClone) and were incubated at 37°C in 5% CO_2_.

### Irradiation Conditions

#### X-Rays

The cells were inoculated in T25 culture flasks 24 h before irradiation, which was subsequently irradiated on X-ray instruments dedicated to radiobiological experiments at the Institute of Modern Physics, Chinese Academy of Sciences. The X-rays were operated at 100 Kev, with a dose rate of 1.0 Gy/min, which source was 0.5 meters away from the sample surface. The cells were irradiated at room temperature.

#### Carbon Ions

The samples were irradiated with the 80 MeV/u carbon ions beam provided by the external tumor treatment terminal of the Lanzhou Heavy Ion Research Facility (HIRFL), and the carbon ions beam provided by HIRFL was calibrated before the irradiation to make sure the LET of the carbon ions irradiated to the sample surface was 30 keV/μm, and the dose rate was 2.0 Gy/min. Irradiation was performed at room temperature. The control samples were sham-irradiated.

### Enzyme-Linked Immunosorbent Assay

The A549/H520/LLC cells in the exponential growth phase were irradiated with 0 Gy, 2 Gy, 4 Gy, 6 Gy X-rays, and Carbon ions irradiation, and the cell culture supernatant was collected at different time points (6, 18, 24, 36, and 48 h), which stored at −4°C for later use. TGF-β, IL-10, HMGB1 ELISA kits were purchased from Neobioscience Technology Co, Ltd. The experimental operation was strictly performed under the instructions.

### Annexin V/PI Double Staining to Detect Cell Apoptosis

Use flow cytometry to detect changes in cell apoptosis after radiation. Collect the overall sample size of 10,000 cells, detect and collect FL-1 (Annexin V-FITC green fluorescence signal) and FL-2 (PI red fluorescence signal) channel information, and use IDEAS Version 6.0 software for analysis. The apoptosis kit was purchased from BD Biosciences Pharmingen.

### Statistical Analysis

The statistical analysis was performed using one-way analysis of variance and an unpaired Student's *t*-test with a 2-tailed distribution, and multiple comparisons have been made. *P* < 0.05 was considered statistically significant. All statistical analyses were performed using IBM's SPSS software (version 20.0).

## Results

### Construction of PPI Network Based on Significantly DEGs in PCA

**Figure 1:**
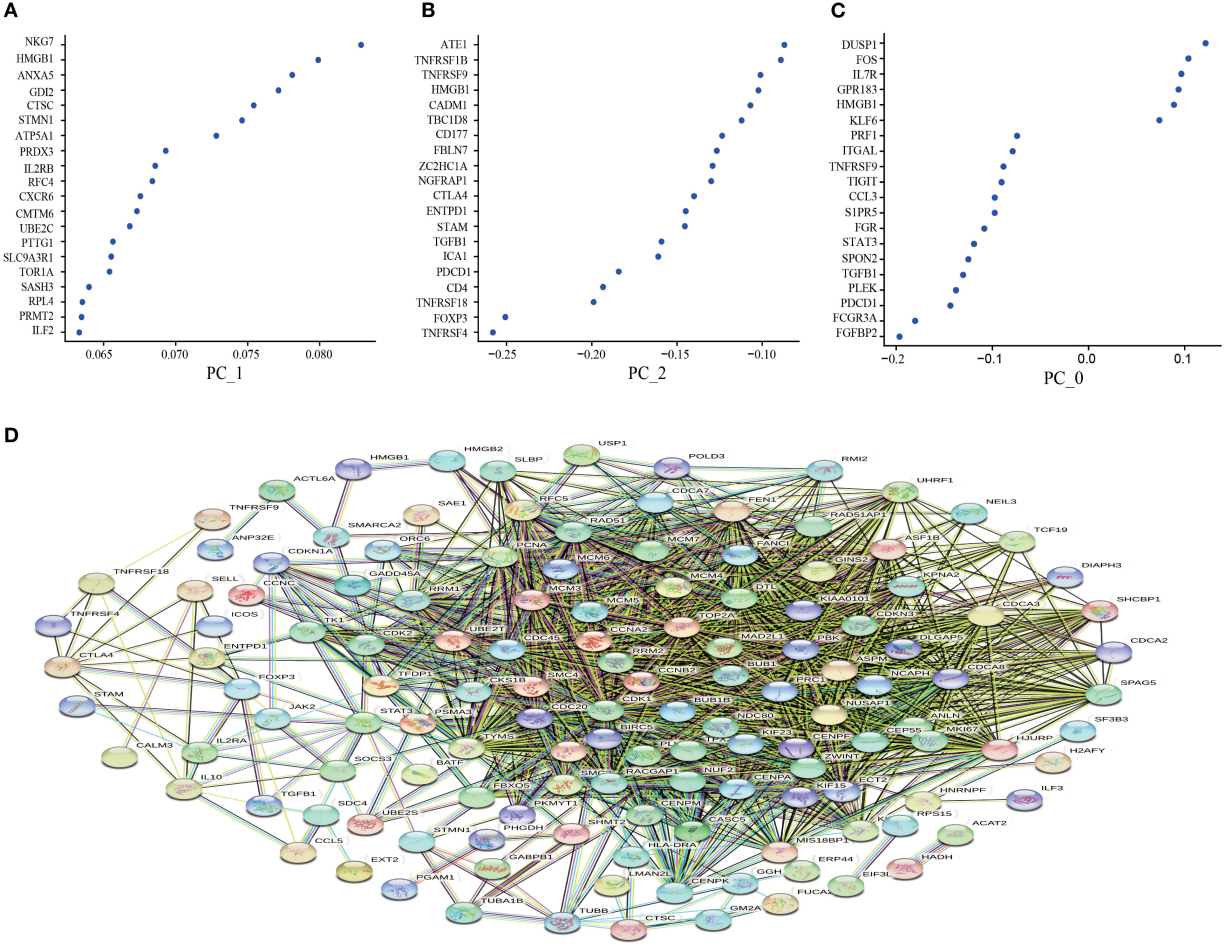
Principal component analysis to screen out the differential genes and construct the protein-protein interaction (PPI) network. **(A)** the resting state (PC_0); **(B)** the activated state (PC_1); **(C)** the non-functional state (PC_2); **(D)** Construction of PPI network based on differential genes from the STRING database. The key modules were determined from the PPI network by the MCODE tool. Co-expression analysis of major marker genes in lung cancer.

Based on the CD8^+^T cell scRNA-seq in lung cancer, we used PCA to screen out the DEGs expressed in the resting state (PC_0), activated state (PC_1), and non-functional state (PC_2) clusters. The larger the absolute value of the numerical value, the more pronounced the gene significance. Interestingly, as a Marker gene in the TME, HMGB1 was significantly enriched in the activated state (Figure 1A), while the immunosuppressive factors TGF-β and IL-10 were significantly enriched in the non-functional state (Figure 1B). In the resting state, these differential genes lacked noticeable distribution differences (Figure 1C). To further clarify the co-expression relationship of marker genes in lung cancer, we constructed a co-expression network based on differential genes from the STRING database and found that almost all independent marker genes have coordinated regulation in the network (Figure 1D).

### The Distribution of Marker Genes in CD8^+^ T Cell Single-Cell Clusters

**Figure 2:**
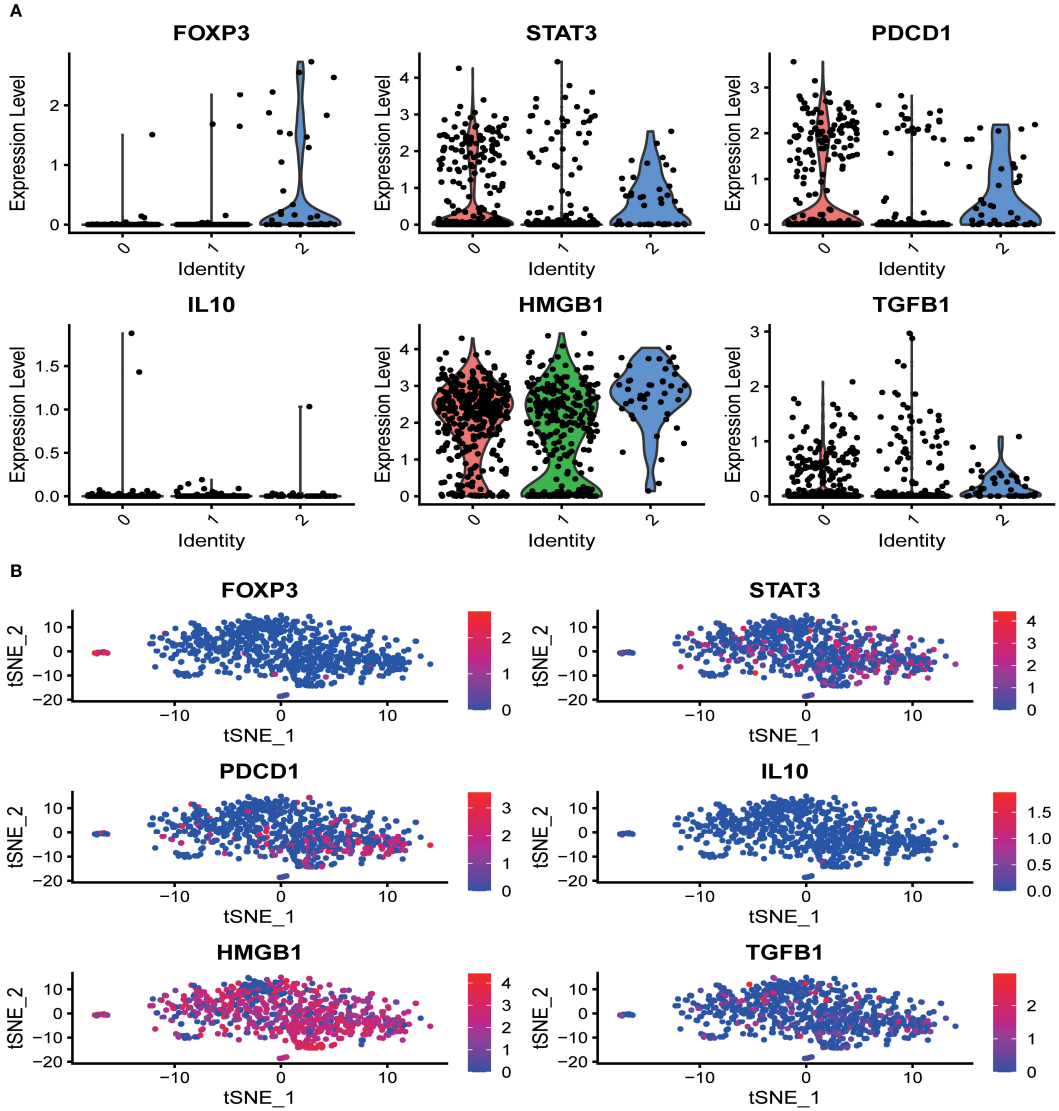
The distribution of marker genes in CD8^+^ T cell single-cell clusters. **(A)** In the different functional states of CD8^+^ T cells, different genes were involved in the expression or enrichment. **(B)** In the activated state of CD8^+^ T cells, the distribution of immune-related genes was significantly different. The immunostimulatory factor HMGB1 was significantly enriched, while the distribution of the immunosuppressive factors was significantly reduced.

Several marker genes, FOXP3, STAT3, PDCD1, TGFB1, IL10, HMGB1, which are significantly related to the tumor microenvironment induced by radiation, had significant functional differences in the distribution of CD8^+^T cells clusters. Irradiation causes the immunogenic death of dying cells, among which the accumulation of HMGBI was pronounced in the resting state and the activated state, while the related inhibitors IL10, TGFB1, FOXP3 (marker genes of Tregs), STAT3 (FOXP3 transcriptional cofactor), and PDCD1 (PD-1 related genes) were significantly enriched in the non-functional state (Figure 2A). We analyzed the distribution trend of the above marker genes in CD8^+^ T cell clusters in the activated state. Interestingly, this phenomenon was still apparent. HMGB1 became the most apparent gene enriched in the activated state of CD8^+^ T cell clusters. The opposite was true for FOXP3 and IL10 (Figure 2B).

### Both X-Rays and Carbon Ions Promoted the Exposure of HMGB1, IL-10, and TGF-βin a Time-Dependent Manner

**Figure 3:**
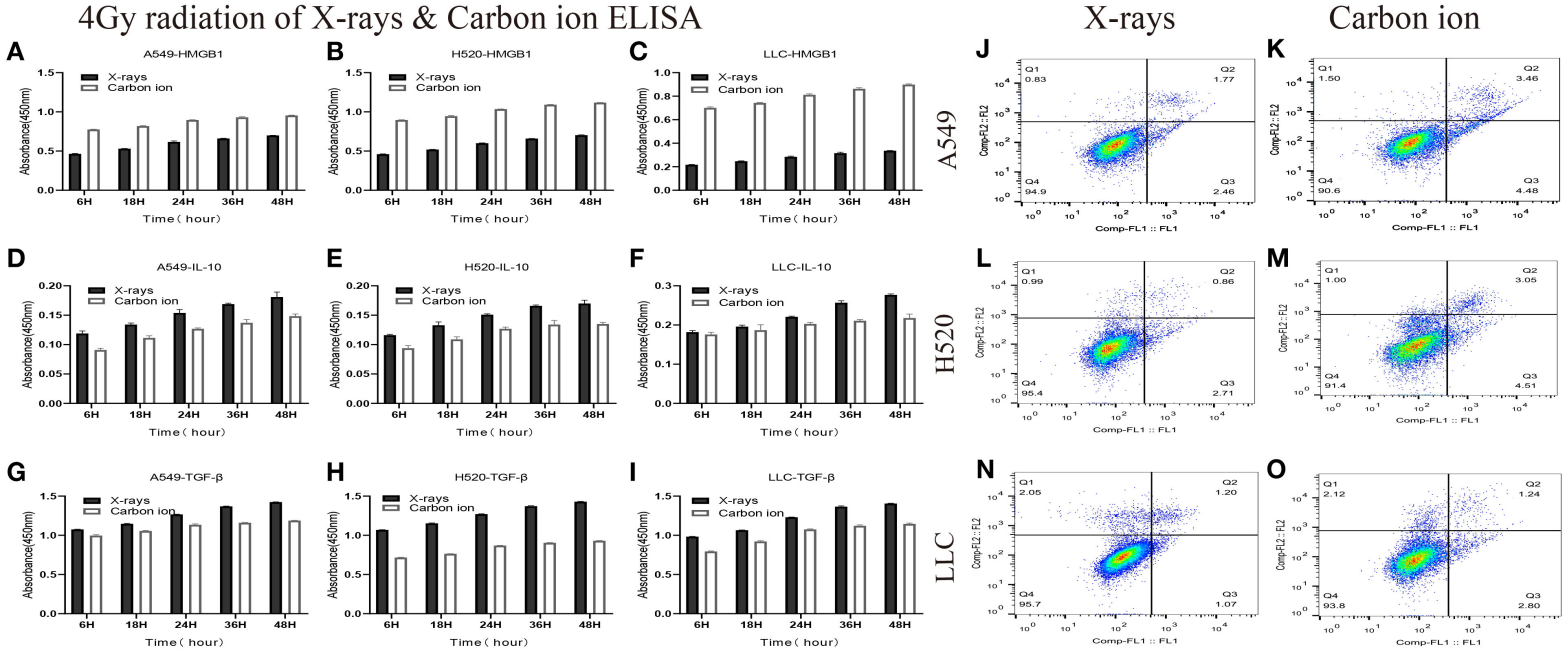
Both X-rays and Carbon ion promoted the exposure of HMGB1, IL-10, and TGF-β in a time-dependent manner **(A–C)** Under 4Gy irradiation (X-rays and Carbon ion), the exposure level of HMGB1 in A549, H520, LLC cell lines. Both types of radiation could induce HMGB1 exposure in a time-dependent manner, and carbon ions significantly increased the exposure level of HMGB1. **(D–F)** The exposure level of IL-10 in A549, H520, LLC cell lines under 4Gy irradiation of X-rays & Carbon ion. **(G–I)** The exposure level of TGF-β in A549, H520, LLC cell lines under 4Gy irradiation. **(J–O)** The differences in apoptosis of A549, H520, LLC cell lines under 4GY irradiation of X-rays and Carbon ion. Under the same physical dose irradiation, carbon ions could significantly increase the apoptosis of the three cell lines than X-rays.

Given the above CD8^+^ T cell cluster scRNA-seq analysis, we found that different immune responses present different immune-related factors. Based on this, we irradiated three different lung cancer cell lines, including human (A549/H520) and murine (LLC), with different physical doses (0, 2, 4, and 6 Gy) X-rays and Carbon ions irradiation, aiming to explore the changing trend of main DAMPs or TAAs after different radiation exposure. Take the exposure levels of HMGB1, IL-10, and TGF-β at different times (6–48 h) in the three cell lines after 4 Gy radiation as an example. It is not difficult to find that the exposure levels of HMGB1, IL-10, and TGF-β in the three cell lines all increased with time in both X-rays and Carbon ions. Interestingly, the exposure level within 18 h after irradiation only slightly increased and reached a peak after 24–36 h, while the main DAMPs and TAAs increased into a plateau after 48 h of irradiation (Figures 3A–I, Table 1). Also, after 48 h of irradiation with 4 Gy X-rays and carbon ions, we analyzed the differences in apoptosis of A549, H520, and LLC cell lines and found that carbon ions can significantly promote cell apoptosis at the same physical dose (Figures 3J–O).

**Table 1:** Time-dependent expression analysis of HMGB1, IL-10 and TGF-β under 4 Gy physical dose radiation (Carbon ions and X-rays) (ng/ml).

**Cells**	**DAMPs**	**6h**	**18h**	**24h**	**36h**	**48h**	***F_***time***_***	***P_***time***_***
A549	HMGB1^a^	4.093, 0.077	4.836, 0.032	5.838, 0.153	6.372, 0.035	6.846, 0.018	399.403	<0.001
	HMGB1^b^	7.730, 0.037^▾^	8.299, 0.076^▾^	9.375, 0.040^▾^	9.863, 0.103^▾^	10.199, 0.063^▾^	472.807	<0.001
H520	HMGB1^a^	4.06, 0.031	4.7, 0.032	5.647, 0.034	6.348, 0.017	6.897, 0.054	2157.176	<0.001
	HMGB1^b^	9.375, 0.04^▾^	10.082, 0.104^▾^	11.445, 0.022^▾^	12.338, 0.069^▾^	12.766, 0.024^▾^	1153.562	<0.001
LLC	HMGB1^a^	19.43, 0.39	22.75, 0.41	27.07, 0.60	30.56, 0.91	33.04, 0.35	379.061	<0.001
	HMGB1^b^	78.789, 1.120^▾^	84.575, 0.506^▾^	94.233, 1.187^▾^	101.484, 1.265^▾^	106.657, 0.858^▾^	557.110	<0.001
A549	${\text{IL-}10}_{\left(\text{pg}/\text{ml}\right)}^{\text{a}}$	0.84, 0.045	0.992, 0.028	1.182, 0.052	1.317, 0.012	1.421, 0.073	51.193	<0.001
	${\text{IL-}10}_{\left(\text{pg}/\text{ml}\right)}^{\text{b}}$	0.490, 0.033^*^	0.715, 0.036^*^	0.868, 0.014^*^	0.962, 0.052^*^	1.224, 0.254	10.790	0.011
H520	${\text{IL-}10}_{\left(\text{pg}/\text{ml}\right)}^{\text{a}}$	0.808, 0.015	0.982, 0.056	1.154, 0.013	1.29, 0.013	1.325, 0.05	76.558	<0.001
	${\text{IL-}10}_{\left(\text{pg}/\text{ml}\right)}^{\text{b}}$	0.525, 0.049^*^	0.689, 0.044^*^	0.868, 0.027^∇^	0.934, 0.066^*^	0.944, 0.026^*^	32.368	0.001
LLC	${\text{IL-}10}_{\left(\text{pg}/\text{ml}\right)}^{\text{a}}$	48.689, 1.397	53.736, 1.297	62.972, 0.612	76.737, 1.935	84.595, 0.971	527.955	<0.001
	${\text{IL-}10}_{\left(\text{pg}/\text{ml}\right)}^{\text{b}}$	47.170, 1.859	51.085, 4.925	56.809, 1.326^▾^	59.729, 1.08^▾^	62.32, 3.594^▾^	17.788	<0.001
A549	TGF-β^a^	0.583, 0.002	0.635, 0.004	0.728, 0.002	0.812, 0.004	0.860, 0.001	3258.027	<0.001
	TGF-β^b^	0.512, 0.008^∇^	0.553, 0.005^∇^	0.609, 0.008^∇^	0.628, 0.003^▾^	0.649, 0.002^▾^	190.477	<0.001
H520	TGF-β^a^	0.578, 0.002	0.640, 0.004	0.731, 0.002	0.815, 0.005	0.864, 0.004	1874.740	<0.001
	TGF-β^b^	0.338, 0.003^▾^	0.363, 0.001^▾^	0.428, 0.002^▾^	0.450, 0.003^▾^	0.471, 0.002^▾^	1550.763	<0.001
LLC	TGF-β^a^	0.836, 0.004	0.924, 0.003	1.110, 0.002	1.270, 0.013	1.321, 0.005	4117.563	<0.001
	TGF-β^b^	0.686, 0.007^▾^	0.821, 0.009^▾^	0.993, 0.008^▾^	1.048, 0.012^▾^	1.077, 0.011^▾^	1134.538	<0.001

a *X-rays*,

b *Carbon ion*,

* *p < 0.05*,

∇ *p < 0.01*,

▾ *p < 0.001 (Represents the comparison between Carbon ion and the X-rays radiation group at the same physical dose and time point)*.

### X-Rays but Not Carbon Ions Increased the HMGB1 Exposure Level in a Dose-Dependent Manner

**Figure 4:**
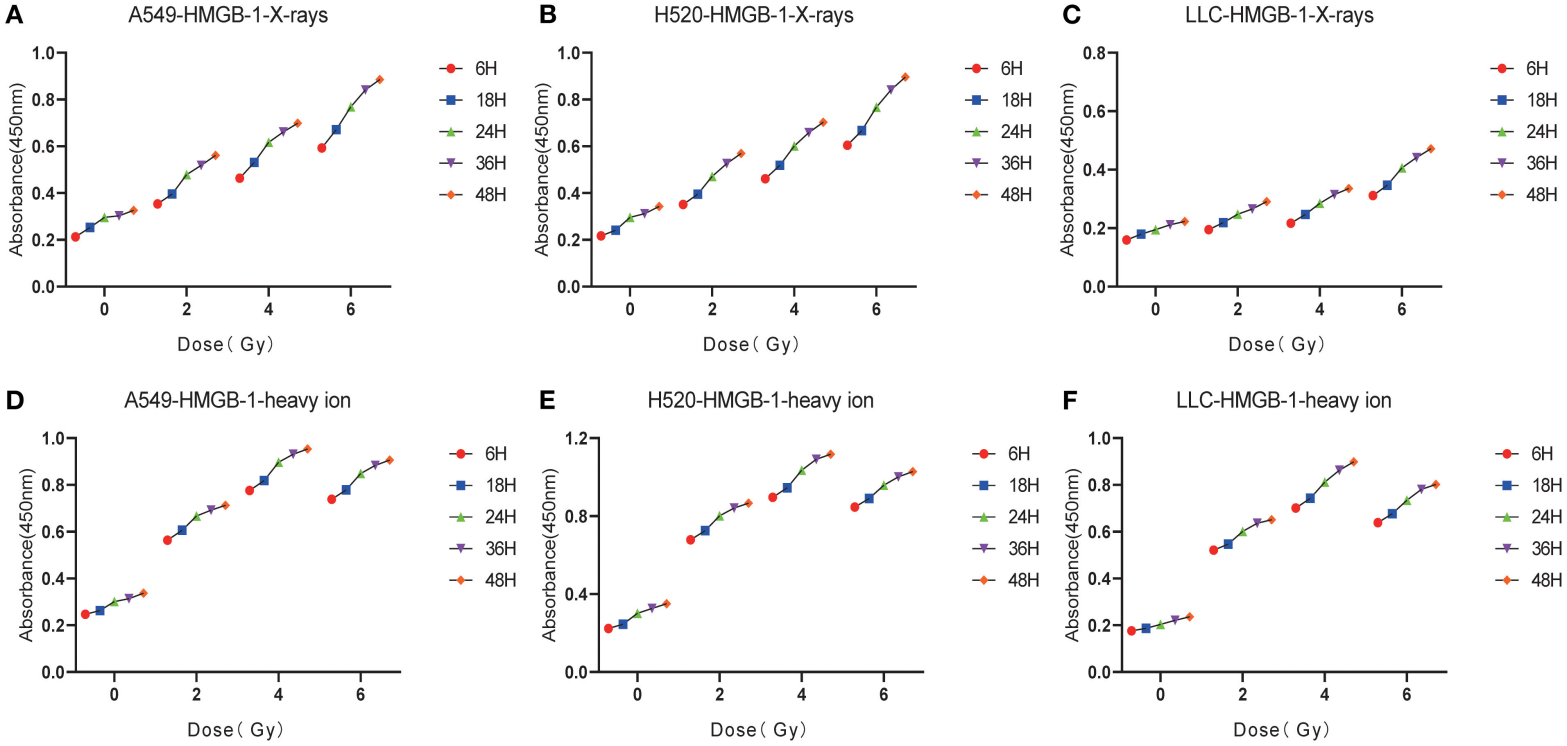
X-rays but not Carbon ion increased the HMGB1 exposure level in a dose-dependent manner. **(A–C)** The exposure level of HMGB1 after 48 hours under different doses (0Gy, 2Gy, 4Gy, 6Gy) of X-rays. As the radiation dose increased, the exposure level of HMGB1 gradually increased. **(D–F)** The exposure level of HMGB1 after 48 hours under different doses (0Gy, 2Gy, 4Gy, 6Gy) of Carbon ions. HMGB1 had the highest exposure level under 4Gy carbon ion irradiation and then entered the shoulder area.

As one of the DAMPs that significantly enhance the anti-tumor immune effect after radiation, the exposure level of HMGB1 is of great significance for the immune surveillance of the tumor microenvironment. After X-rays and Carbon ions irradiation with the same physical dose (2–6 Gy), the exposure of HMGB1 showed different trends. For X-rays, the exposure level of HMGB1 showed a dose-dependent increase, but the increase was limited in the low dose (0-2 Gy) range, and a substantial increase was showed after 4 Gy. Interestingly, the exposure level of HMGB1 caused by Carbon ions irradiation peaked at a physical dose of 4 Gy and then slowly decreased. Besides, under the same physical dose, the exposure level of HMGB1 caused by Carbon ions irradiation was significantly higher than that of X-rays. There was no significant difference in the above trend among the three lung cancer cell lines (Figure 4, Table 2).

**Table 2:** Dose-dependent expression analysis of HMGB1 after 48 h of carbon ions and X-rays radiation (ng/ml).

**Cells**	**Radiation**	**0 Gy**	**2 Gy**	**4 Gy**	**6 Gy**	***F_***dose***_***	***P_***dose***_***
A549	X-rays	2.664, 0.014	5.173, 0.089	6.846, 0.018	9.338, 0.120	2732.034	<0.001
	C-ion	2.764, 0.055	6.925, 0.071^∇^	10.201, 0.062^▾^	9.503, 0.020	7326.635	<0.001
H520	X-rays	2.832, 0.028	5.283, 0.016	6.897, 0.054	9.494, 0.002	14377.758	<0.001
	C-ion	2.901, 0.014	8.954, 0.039^▾^	12.765, 0.023^▾^	11.336, 0.063^▾^	22818.361	<0.001
LLC	X-rays	20.08, 0.40	27.76, 0.21	33.04, 0.35	49.77, 0.64	3442.307	<0.001
	C-ion	20.69, 0.46	71.99, 0.63^▾^	106.66, 0.86^▾^	92.82, 0.36^▾^	15343.844	<0.001

∇ *p < 0.01*,

▾ *p < 0.001 (Represents the comparison between Carbon ion and the X-rays radiation group at the same physical dose and time point)*.

### Low-Dose Irradiation Is More Likely to Cause the Enrichment of Immunosuppressive Factors

**Figure 5:**
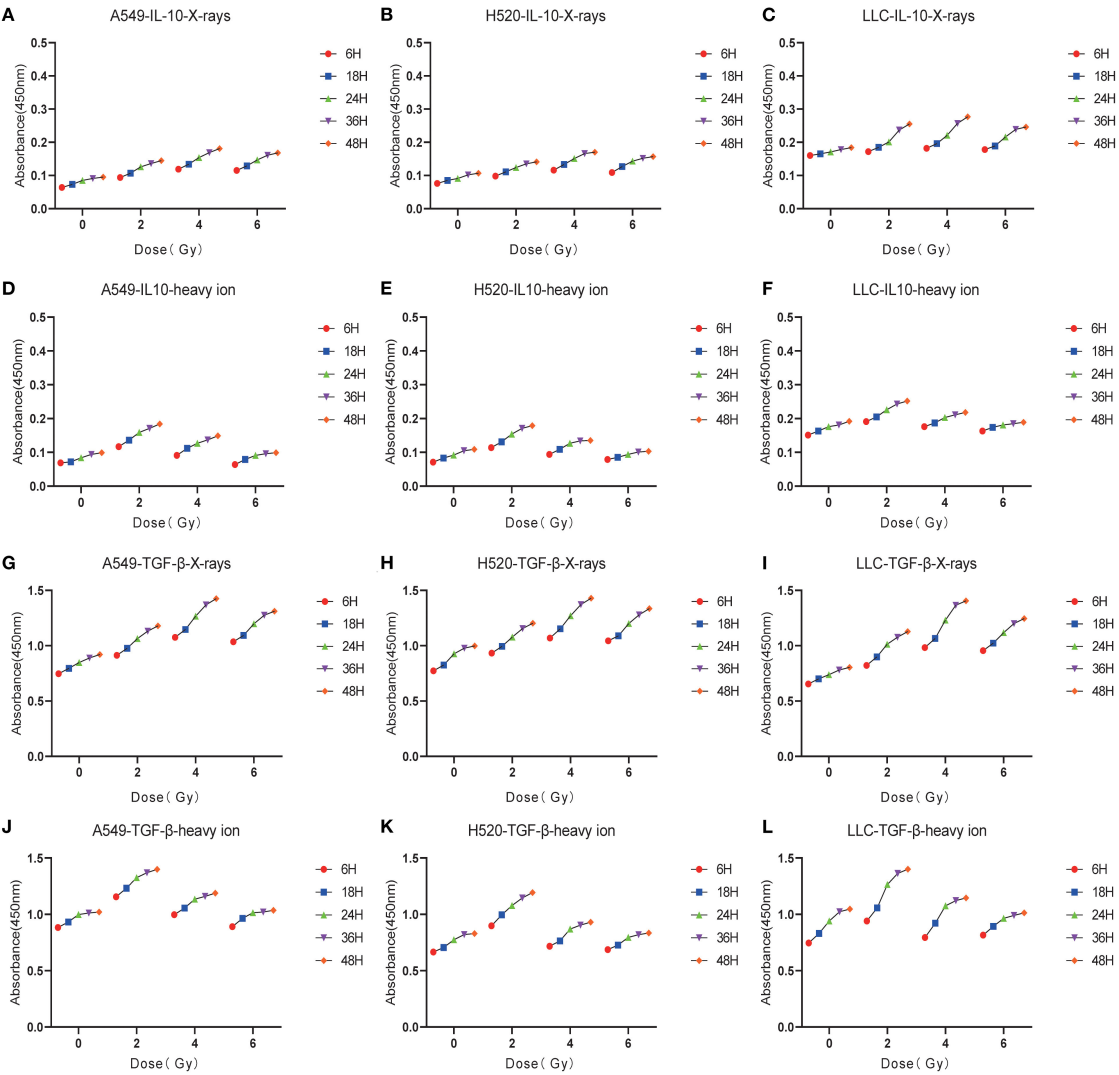
Low-dose irradiation was more likely to cause the enrichment of immunosuppressive factors such as IL-10, TGF-β. **(A–C)** The exposure level trend of IL-10 under different doses of X-rays. **(D–F)** The exposure level of IL-10 under different doses of Carbon ions. **(G–I)** The exposure level trend of TGF-β under different doses of X-rays. **(J–L)** The exposure level trend of TGF-β under different doses of Carbon ions.

DAMPs or TAAs released by dying tumor cells caused by radiation include not only immune enhancing elements such as calreticulin and HMGB1 but also immunosuppressive factors such as IL-10 and TGF-β. In this study, we focused on analyzing the changing trends of IL-10 and TGF-β caused by X-rays and Carbon ions radiation. For X-rays, compared with the non-irradiated group, the physical dose at which the IL-10 exposure level reaches the peak was 4 Gy. As the dose increases, the exposure gradually decreased. The above trend also existed in the changes in TGF-β exposure levels. However, the peak exposure of IL-10 and TGF-β in the Carbon ions radiation group was a physical dose of 2 Gy. Interestingly, this trend was consistent with the RBE value of Carbon ions radiation (Figure 5, Table 3).

**Table 3:** Dose-dependent expression analysis of IL-10&TGF-β after 48 h of carbon ions and X-rays radiation (ng/ml).

**Cells**	**DAMPs**	**0Gy**	**2Gy**	**4Gy**	**6Gy**	***F_***dose***_***	***P_***dose***_***
A549	IL-10^a^^(pg/ml)^	0.568, 0.035	1.098, 0.013	1.421, 0.073	1.308, 0.013	166.986	<0.001
	IL-10^b^^(pg/ml)^	0.582, 0.031	1.370, 0.047^*^	1.067, 0.032^*^	0.581, 0.063^∇^	19.547	0.007
H520	IL-10^a^^(pg/ml)^	0.709, 0.032	1.060, 0.027	1.325, 0.05	1.209, 0.026	117.103	<0.001
	IL-10^b^^(pg/ml)^	0.689, 0.029	1.329, 0.024^∇^	0.944, 0.026^*^	0.625, 0.031^∇^	268.125	<0.001
LLC	IL-10^a^^(pg/ml)^	49.402, 0.773	75.958, 1.383	84.595, 0.971	72.477, 1.75	554.724	<0.001
	IL-10^b^^(pg/ml)^	48.928, 1.553	75.108, 2.387	62.32, 3.594^▾^	51.768, 1.268^▾^	88.973	<0.001
A549	TGF-β^a^	0.476, 0.003	0.659, 0.003	0.86, 0.001	0.765, 0.002	6896.873	<0.001
	TGF-β^b^	0.474, 0.004	0.818, 0.003^▾^	0.649, 0.002^▾^	0.537, 0.001^▾^	7207.095	<0.001
H520	TGF-β^a^	0.528, 0.001	0.678, 0.002	0.864, 0.005	0.784, 0.005	3219.170	<0.001
	TGF-β^b^	0.532, 0.001	0.653, 0.006^*^	0.468, 0.002^▾^	0.406, 0.001^▾^	1908.329	<0.001
LLC	TGF-β^a^	0.655, 0.002	0.993, 0.002	1.321, 0.005	1.126, 0.007	14028.502	<0.001
	TGF-β^b^	0.652, 0.002	1.401, 0.007^▾^	1.077, 0.011^▾^	0.922, 0.008^▾^	6552.511	<0.001

a *X-rays*,

b *Carbonion*,

* *p < 0.05*,

∇ *p < 0.01*,

▾ *p < 0.001 (Represents the comparison between Carbon ion and the X-rays radiation group at the same physical dose and time point)*.

## Discussion

Nowadays, more and more studies on radiotherapy causing abscopal effects and participating in anti-tumor immune response indicate that inducing immunogenic changes has become one of the essential mechanisms for radiotherapy to exert immune synergy (34). Based on this, a variety of approaches to enhance the immunogenicity of apoptotic cells have been developed (7, 35). Conventional X-rays-induced immunogenicity changes in tumor cells have been reported. However, due to the limitations of radiotherapy resistance, the immune synergy of radiotherapy needs to be further improved (36). Heavy ions have become definitive radiation therapy due to their superior radiobiological effects (23, 37). However, the advantages and specific mechanisms of the immunogenic changes induced by heavy ions in dying tumor cells are still unclear.

Tumor immunotherapy is an anti-tumor immune response driven by T cells (38). Since the induction of cytotoxic T cells depends on the activation and maturation of DC (39), the research on ICD mainly focuses on the DC-T cell axis and the primary markers for detecting ICD (14, 19). In this study, based on the PCA of scRNA-seq data of lung cancer CD8^+^T cell clusters, the DEGs involved in different immune response stages were analyzed, and the main marker genes were screened out. In this study, we evaluated and compared the exposure levels of the relevant antigens of the three lung cancer cell lines under conventional X-rays and carbon ions radiation, which provided a reference for future heavy ion-induced immunogenicity changes and immune regulation.

HMGB1 plays a vital role in ICD and inducing an anti-tumor immune response (40). HMGB1 binds to TLR-4 and receptors to form advanced glycosylation end products, which promote the production of cytokines, cross-presentation of related antigens, and the maturation and activation of DC cells, thereby activating helper T cells and effector T cells (41, 42). Based on the scRNA-seq data of CD8^+^T cell clusters, we found that the accumulation of HMGB1 was particularly significant in the immune effector clusters of CD8^+^T cells. In contrast, the accumulation of TGF-β, IL-10, FOXP3, and STAT3 were mainly concentrated in the immunosuppressive state (43). During tumorigenesis and progression, the uptake of apoptotic cells by surrounding macrophages is accompanied by the release of anti-inflammatory signals such as TGF-β (44). Therefore, apoptosis can promote tumor tolerance (45). To a certain extent, the exposure of these immunosuppressive factors also provides a basis for explaining radiotherapy resistance or tolerance (46). As we know, the form of cell death caused by carbon ions is different from X-rays, and its higher LET and unique brag peak have become one of the advantages of replacing traditional X-rays (47). Research by OnishiM et al. showed that the exposure level of HMGB1 increased with the linear energy transfer (LET) value (48). Yutaka Takahashi et al. found that the exposure level of HMGB-1 in the cell culture supernatant collected 48 h after carbon ions irradiation increased by more than three times compared with untreated cells (5). Also, the effect of carbon ion radiation on ICD can spread to the peripheral blood (49). Although previous studies have shown that X-ray irradiation and chemotherapy can induce ICD (50), there are few studies on the exposure differences and trends of related antigens that play different roles in the tumor immune response stage under different irradiations.

Similar to the increase in other DAMPs, such as calreticulin after irradiation, the exposure of HMGB1 showed a time and dose-dependent relationship to a certain extent. Under the same physical dose of X-ray and carbon ion irradiation, the exposure level of HMGB1 showed a time-dependent trend, which was different in X-rays and carbon ions. Specifically, the exposure level of HMGB1 induced by carbon ions was higher than that of X-rays, especially in mouse Lewis cells. Interestingly, the exposure level of HMGB1 increased insignificantly within 18 h after irradiation but increased significantly within 24–36 h, and the shoulder area appeared within 48 h. Similar to our research, Yangle Huang et al. found that the three types of irradiation of photons, protons, and carbon ions also increased the exposure of surface-exposed calreticulin (ecto-CRT) in a time-dependent manner (51). At 48 h after irradiation, ecto-CRT exposure increased significantly but only slightly increased in various tumor cell lines at 12 h after irradiation.

At the same time, after irradiation, the exposure level of HMGB1 induced by X-rays but not carbon ions irradiation was dose-dependent. Under the 6 Gy physical dose of carbon ions irradiation, the exposure level of HMGB1 tended to be flat or even lower, which was similar to the research conclusions of Yangle Huang et al. Cell death caused by apoptosis may be highly immunogenic. In contrast, the immunogenicity of necrotic cells may be lower than that of cells undergoing immune apoptosis (41, 52). Based on this, we speculate that carbon ions irradiation with 6 Gy physical dose may cause some other types of death pattern, and the specific mechanism that needs to be further studied. Also, studies (24) have shown that even in normal cells, radiation with a dose between 4 and 12 Gy can induce cytoplasmic HMGB1 translocation and stimulate the time and dose-dependent release of HMGB1 *in vivo* and *in vitro*. In the dose range of 4 to 8 Gy, the release of HMGB1 was induced as early as 6 h after stimulation.

Radiation induces ICD of tumor cells to activate M2 macrophages and then secrete various cytokines, including TGF-β and IL-10 (29, 53). TGF-β is a potent immunosuppressive factor in TME, which can damage the function of DCs and inhibit the activation of T cells and promote the transformation of naive CD4^+^T cells into Treg cells (54). TGF-β is usually secreted in the form of inactivation in the extracellular matrix and is released from the latency-related peptide (LAP) by external stimuli such as radiation. Besides, the production of reactive oxygen species (ROS) after radiation can also promote the release of TGF-β, thus increasing the immunosuppressive effect (25). Similarly, IL-10, an important immunosuppressive factor, induces an immunosuppressive pathway by promoting S100A9 nuclear localization and MDSC maturation (55). Consistent with HMGB1, the exposure levels of IL-10 and TGF-β under X-rays and carbon ions irradiation were also time-dependent. Interestingly, the exposure levels of IL-10 and TGF-β induced by different physical doses of X-rays and carbon ions showed different trends. Under 4 Gy X-rays irradiation, the exposure level of IL-10 and TGF-β reached a peak and then entered a plateau or decline phase. However, this trend appeared under 2 Gy carbon ions irradiation. Studies have shown that low-dose radiation may increase the expression of immunosuppressive factors or immune checkpoint molecules. Besides, low-dose radiation may also activate immune suppression and angiogenesis (46) and promote M2 macrophages to inhibit the anti-tumor response and promote metastasis by producing arginase and cytokines TGF-β and IL-10 (56). Therefore, the above data also provided a reference for immune tolerance induced by low-dose irradiation. This study also found that carbon ions above 4 Gy could significantly reduce the exposure level of immunosuppressive factors IL-10 and TGF-β. Interestingly, at this dose, carbon ions radiation-induced the peak exposure level of HMGB1, which suggested that 4 Gy radiation of carbon ions may reach an ideal balance point in promoting immune effects and reducing immune tolerance since this study was only carried out on lung cancer cell lines *in vitro*, which needs to be further verified by animal-related experiments and clinical experiments.

In summary, by comparing the exposure levels of X-rays and carbon ions radiation to DAMPs or TAAs involved in the immune response, we found that both X-rays and carbon ions can change the immunogenicity of lung cancer cells in a time-dependent manner. Based on further analysis of the “time window” and “dose window” of carbon ions radiation, it was found that carbon ions may be more advantageous than traditional X-rays in terms of inducing immunogenic changes. Based on this finding, further exploration of two kinds of radiation-induced immunogenicity and TME changes in mouse tumor-bearing models will be our next task.

## Data Availability Statement

The original contributions presented in the study are included in the article/supplementary material, further inquiries can be directed to the corresponding author/s.

## Author Contributions

JR: conceptualization, methodology, software, formal analysis, validation, data curation, writing–original draft, writing–review and editing, visualization. JW: methodology, software, formal analysis, writing-original draft. YM and CZ: methodology, validation, formal analysis, investigation. ZD and JG: data curation, investigation. QG: conceptualization, writing-review and editing, supervision, project administration. All authors: contributed to the article and approved the submitted version.

## Conflict of Interest

The authors declare that the research was conducted in the absence of any commercial or financial relationships that could be construed as a potential conflict of interest.
